# The effect of a test ordering software intervention on the prescription of unnecessary laboratory tests - a randomized controlled trial

**DOI:** 10.1186/s12911-017-0416-6

**Published:** 2017-02-20

**Authors:** Carlos Manuel Silva Martins, Andreia Sofia da Costa Teixeira, Luís Filipe Ribeiro de Azevedo, Luísa Maria Barbosa Sá, Paulo Alexandre Azevedo Pereira Santos, Maria Luciana Gomes Domingues do Couto, Altamiro Manuel Rodrigues da Costa Pereira, Alberto Augusto Oliveira Pinto Hespanhol, Cristina Maria Nogueira da Costa Santos

**Affiliations:** 1 Family Medicine Unit, Social Sciences and Health Department of the Faculty of Medicine of Porto, Porto, Portugal; 2 DCC- Faculty of Sciences, SQIC at Institute of Telecommunications, University of Porto, Porto, Portugal; 3 Centre for Research in Health Technologies and Information Systems (CINTESIS), Information Sciences and Decision on Health Department (CIDES), Faculty of Medicine, University of Porto, Porto, Portugal; 4 Unidade de Medicina Geral e Familiar, Faculdade de Medicina da Universidade do Porto, Al. Prof. Hernâni Monteiro, 4200-319 Porto, Portugal

**Keywords:** Preventive health services, Primary health care, Evidence-based practice, Decision support systems, clinical, Decision making, computer-assisted

## Abstract

**Background:**

The way software for electronic health records and laboratory tests ordering systems are designed may influence physicians’ prescription. A randomised controlled trial was performed to measure the impact of a diagnostic and laboratory tests ordering system software modification.

**Methods:**

Participants were family physicians working and prescribing diagnostic and laboratory tests.

The intervention group had a modified software with a basic shortcut menu changes, where some tests were withdrawn or added, and with the implementation of an evidence-based decision support based on United States Preventive Services Task Force (USPSTF) recommendations. This intervention group was compared with usual software (control group).

The outcomes were the number of tests prescribed from those: withdrawn from the basic menu; added to the basic menu; marked with green dots (USPSTF’s grade A and B); and marked with red dots (USPSTF’s grade D).

**Results:**

Comparing the monthly average number of tests prescribed before and after the software modification, from those tests that were withdrawn from the basic menu, the control group prescribed 33.8 tests per 100 consultations before and 30.8 after (*p* = 0075); the intervention group prescribed 31.3 before and 13.9 after (*p* < 0001). Comparing the tests prescribed between both groups during the intervention, from those tests that were withdrawn from the basic menu, the intervention group prescribed a monthly average of 14.0 vs. 29.3 tests per 100 consultations in the control group (*p* < 0.001). From those tests that are USPSTF’s grade A and B, intervention group prescribed 66.8 vs. 74.1 tests per 100 consultations in the control group (*p* = 0.070). From those tests categorised as USPSTF grade D, the intervention group prescribed an average of 9.8 vs. 11.8 tests per 100 consultations in the control group (*p* = 0.003).

**Conclusions:**

Removing unnecessary tests from a quick shortcut menu of the diagnosis and laboratory tests ordering system had a significant impact and reduced unnecessary prescription of tests.

The fact that it was not possible to perform the randomization at the family physicians’ level, but only of the computer servers is a limitation of our study. Future research should assess the impact of different tests ordering systems during longer periods.

**Trial registration:**

ISRCTN45427977, May 1^st^ 2014 (retrospectively registered).

## Background

Informatics has undoubtedly changed the way societies live, socialize, learn, work, and deal with healthcare. We now live in a period of increasing concern about the excessive presence of medicine in our lives [1–3]. When inefficient software is combined with a nonevidence-based medical practice, there is the risk of patient harm, significant impact to quality of life, and damage to the healthcare system due to unnecessary costs.

The implementation of electronic health records (EHR) has both potential benefits and drawbacks [4]. Among the benefits, the prevention of medical errors and the promotion of patient safety has often been mentioned and confirmed in clinical practice [4–6]. Despite the positive effects of EHR implementation in clinical practice, a range of barriers faced by physicians has been identified. These barriers may include technical and financial aspects, time, psychological, social, legal, and organizational changes to the process [7]. After having removed the first barriers to EHR implementation, it is now time to implement continuing improvement and development of the available tools and to incorporate the scientific evidence obtained to this point [4, 8–10].

To achieve better patient safety standards and improve healthcare system cost-effectiveness, there has been a worldwide effort to implement an integrated EHR system with diagnostic and laboratory test ordering communication systems [11–13]. There have also been attempts to incorporate clinical decision support systems to further improve the quality of medicine. Prescribing diagnostic and laboratory tests is a key component of medical consultation. In the primary health care setting, tests are often ordered with preventive intentions and fulfillment of patient expectations [14, 15]. There is also great uncertainty and variability among family physicians’ ordering routines [16–18]. The effects of test ordering communication systems integrated with clinical decision support systems have been reported in various clinical practice settings. Main C et al. (2010) have performed a systematic review of this topic and reported that integration of clinical decision support systems resulted in significant benefits to the prescribing process and practitioner performance outcomes in nearly two-thirds of the 24 studies that met the inclusion criteria [19].

**Fig. 1:**
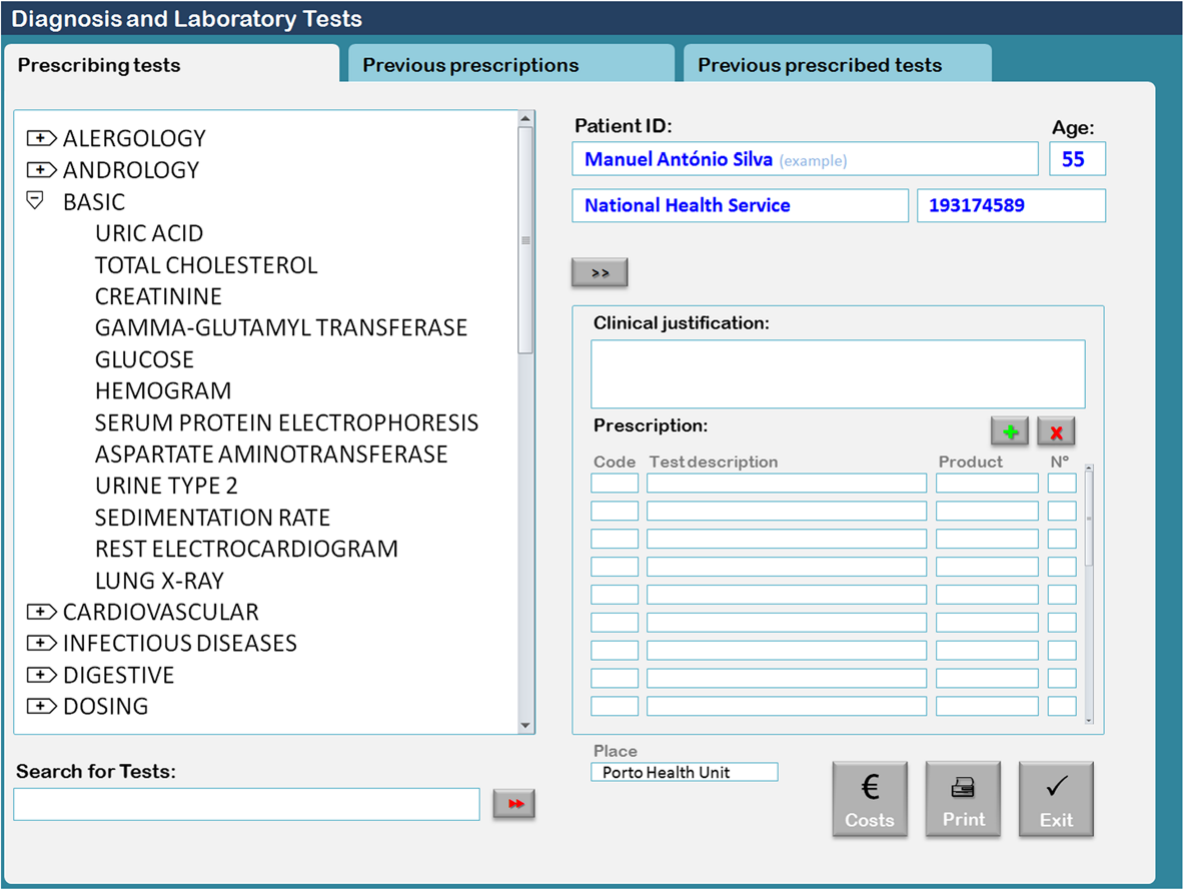
Usual ordering communication system: the basic shortcut menu

In Portugal, the use of EHR software with a diagnostic and laboratory test order communication system has been mandatory since September 2011. Most of the primary healthcare centers use software called *Sistema de Apoio ao Médico* (Physician’s Support System [SAM]). In the module used to order diagnostic and laboratory tests, physicians access a searchable test menu by two possible strategies: 1) typing the test name in a search box or 2) browsing by a shortcut menu structure (Fig. 1). Different menus are available for most areas of medicine, including basic, allergology, andrology, cardiovascular, Infectious diseases, digestive, dosing, endocrinology, gynaecology, haematology, rheumatology, obstetrics, oncology, otorhinolaringology, osteoarticular, preoperative, respiratory, central nervous system, urology and nephrology. Under each menu there is a set of specific lab tests. Physicians can choose one or more tests by double-clicking each test or can choose the entire set by double-clicking on the shortcut menu’s title. For example, the basic menu is composed of uric acid, total cholesterol, creatinine, gamma-glutamyl transferase, glucose, hemogram, serum protein electrophoresis, aspartate aminotransferase, urine type 2, sedimentation rate, electrocardiogram, and lung X-ray tests.

If a physician would like to choose only hemogram and glucose, they must double-click on each test. However, if they would like the entire basic set of tests, they must double-click on the basic menu title. Our research team suspected that this basic shortcut menu is often selected during routine consultations in which patients ask for routine check-ups without specific reason. As we have shown in a previous study, there is a high prevalence of Portuguese adults (99.2%) that believe they should have routine blood and urine tests annually [14]. This statistic demonstrates the importance of examining the effectiveness and efficiency of this basic submenu.

Through a randomized controlled trial, the primary aim of the present study was to compare the effects of modifying the EHR ordering communication system (modified SAM) by changing the basic shortcut menu and adding a clinical decision support system based on the integration of the United States Preventive Services Task Force (USPSTF) recommendations. After the last primary healthcare reform in Portugal, primary healthcare centers have been divided into healthcar center groups [20]. In a healthcare center group, the informatics network is linked through servers that may serve more than one healthcare center. Creating a modified version of the SAM software requires it to be installed at a server level, which determines that all physicians at all healthcare centers served by that server will receive the same version of the software. For this reason, it was not possible to randomize the study at the physician level. Rather, we had to randomize the servers at the healthcare center group level.

## Methods

### Trial design

All servers of the Western Oporto grouping of health centers counted for randomization, except that serving the center where study authors worked (to avoid possible bias). The remaining nine servers were randomized into two groups: 1) five servers were randomly allocated to the intervention group and 2) four servers to the control group.

### Participants

All family physicians working and prescribing diagnostic and laboratory tests in the Western Oporto group of health centers (except in those where the authors worked) participated in this study.

Data of the diagnostic and laboratory tests prescriptions were centrally collected by informatics staff belonging to the Ministry of Health and sent to the research team without patients’ or physicians’ identifications.

### Interventions

**Fig. 2:**
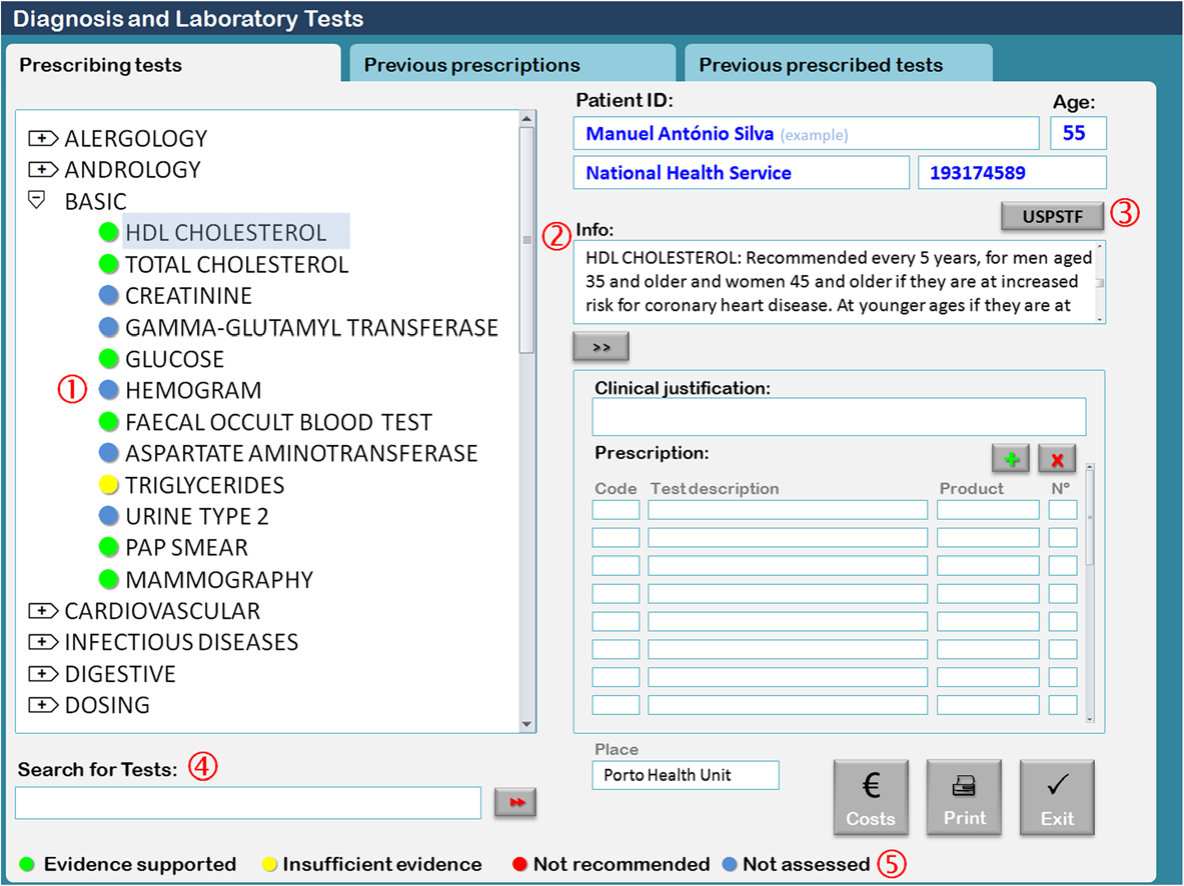
Modified ordering communication system: the basic shortcut menu. *Red numbers*: 1- Traffic lights coloured dots according to United States Preventive Services Task Force recommendations grades. 2- Text box with the summary of the recommendation for each selected test. 3-Link to the original recommendation at the USPSTF’s website. 4-Search for tests box, where any test, including those removed from the basic menu, can be searched and requested by typing the test’s name. 5- Legend of the coloured dots

The control group continued to use the standard version of the EHR software (SAM) as presented in Fig. 1. The intervention group used a modified version of the software (SAM modified) installed in each server (Fig. 2). Software modifications consisted of two principal changes: 1) Basic shortcut menu changes; the composition of the basic menu set of diagnostic laboratory tests was changed. Some tests were removed, including uric acid, serum protein electrophoresis, sedimentation rate, electrocardiogram, and lung X-ray. Other tests were added, including HDL cholesterol, faecal occult blood test, triglycerides, Pap smear, and mammography. Although some tests were removed from the basic menu, physicians were still able to request them by typing their names in the search for tests box. 2) Addition of an evidence-based decision support. For the tests listed in Table 1, we added traffic light-based coloured dots according to the USPSTF recommendations and an additional information box containing the summary of the USPSTF recommendation and a link to the integral recommendation at the USPSTF website (Fig. 2) [21].

**Table 1:** United States Preventives Services Task Force (USPSTF) recommendations (March, 2012)

Coloured dots	Grade^a^	Test	Summary of the recommendations
Red	D	Pelvic ultrasound	Not recommended as routine screening for ovarian cancer.
Red	D	Cancer antigen 19-9	Not recommended as routine screening for pancreatic cancer.
Red	D	Rest electrocardiography	Not recommended in asymptomatic adults at low risk for coronary heart disease events.
Red	D	Exercise electrocardiography	Not recommended in asymptomatic adults at low risk for coronary heart disease events.
Red	D	Carotid artery ultrasound	Not recommended as screening for asymptomatic carotid artery stenosis in the general adult population.
Red	D	Spirometry	Not recommended as screening adults for chronic obstructive pulmonary disease.
Red	D	Hepatitis B surface antigen	Not recommended as routine screening.
Red	D	Hepatitis C antibodies	Not recommended as routine screening.
Yellow	I	Triglycerides	There is currently insufficient evidence of the benefit of including triglycerides as a part of the initial tests used to screen routinely for dyslipidemia.
Yellow	I	Prostate-specific antigen	The evidence is insufficient to recommend for or against routine screening for prostate cancer in men younger than 75.
Yellow	I	Lung computed tomography	The evidence is insufficient to recommend for or against routine screening for lung cancer.
Yellow	I	Lung X-ray	The evidence is insufficient to recommend for or against routine screening for lung cancer.
Yellow	I	Thyroid-stimulating hormone	The evidence is insufficient to recommend for or against routine screening.
Green	B	Glucose	Screening for type 2 diabetes recommended in asymptomatic adults with sustained blood pressure (either treated or untreated) greater than 135/80 mm Hg.
Green	A/B	Total cholesterol	Recommended every 5 years, for men aged 35 and older and women 45 and older if they are at increased risk for coronary heart disease. At younger ages if they are at increased risk for coronary heart disease.
Green	B	Mammography	Biennial screening recommended for women aged 50 to 74 years.
Green	A	Cervicovaginal cytology	Every 3 years screening recommended for women who have cervix, 21 to 65 years old.
Green	A	Faecal occult blood test	Recommended annually as a possible method of screening for colorectal cancer, 50 to 75 years.
Green	A	Colonoscopy	Recommended every 10 years as a possible method of screening for colorectal cancer, 50 to 75 years.
Green	A	Flexible sigmoidoscopy	Recommended every 5 years as a possible method of screening for colorectal cancer, 50 to 75 years.
Green	A/B	HDL cholesterol	Recommended every 5 years, for men aged 35 and older and women 45 and older if they are at increased risk for coronary heart disease. At younger ages if they are at increased risk for coronary heart disease.
Green	B	DXA^b^ of the hip and lumbar spine	Screening recommended for osteoporosis in women aged 65 years or older and in younger women whose fracture risk is equal to or greater than that of a 65-year-old.
Green	A	Venereal Disease Research Laboratory	Recommended for persons at increased risk for syphilis infection.

^a^USPSTF grades A and B: the USPSTF recommends the service, marked with green dots. Grade D: the USPSTF recommends against the service, marked with red dots. Grade I: the USPSTF concludes that the current evidence is insufficient to assess the balance of benefits and harms of the service, marked with yellow dots

^b^ *DXA* Dual-energy X-ray absorptiometry

The selection of the specific tests to be removed from or added to the basic shortcut menu was made after discussion and debate with eight family physicians selected by proximity and convenience. The main rational was to remove tests that are not recommended to be used as routine screening tools and to substitute them with others that may be recommended for certain risk groups. However, creating a change that was that was not too disruptive to the current family physicians’ practices was a concern, and that is why tests such as creatinine, gamma-glutamyl transferase, hemogram, aspartate aminotransferase, and urine type 2 were left in the basic shortcut menu.

EHR software modifications were implemented on 30^th^ and 31^st^ May 2012 in all of the intervention groups’ servers. Prospective monthly monitoring and data collection occurred until 31^st^ January 2013. To allow a pre-post analysis in both groups, a retrospective monthly data collection of both control and intervention groups was also performed between 1^st^ December 2011 and 31^st^ May 2012.

The collected data included monthly parameters, including numbers of prescribing family physicians, face to face consultations made, and number of each prescribed diagnostic and laboratory test.

### Independent variables

Group (control and intervention) and time (before and after the modifications) were the independent variables.

### Dependent variables

Primary outcomes were chosen to assess the impact of our intervention on the number of diagnostic and laboratory tests prescribed by physicians using four different perspectives: 1) impact on the number of the prescriptions of diagnostic and laboratory tests that were withdrawn from the basic menu; 2) impact on the number of the prescriptions of diagnostic and laboratory tests that were added to the basic menu; 3) impact on the number of the prescriptions of diagnostic and laboratory tests that were marked with green dots (USPSTF recommendation grades A and B); and 4) impact on the number of prescriptions of diagnostic and laboratory tests that were marked with red dots (USPSTF recommendation grade D). Diagnostic and laboratory tests were the dependent variables.

### Sample size

Given administrative and technical barriers related to the permission process for the introduction of EHR software modifications, our sample size was obtained by convenience. We decided to run this trial at the Western Oporto grouping of healthcare centers, which includes a total of 15 primary care healthcare centers and an informatics network of 10 servers. One of the servers, which served the healthcare center in which some of the study authors worked, was excluded from the study.

The power calculation was done for the mean number of monthly prescribed tests per 100 consultations comparison between control and intervention groups. A significance level of 0.05, a power of 0.8, and a standard deviation of two tests prescribed per 100 consultations per month was considered. A mean difference of 3.3 tests prescribed per 100 consultations per month between control and intervention groups was used.

### Randomization

The remaining nine servers were sequentially numerated and randomly assigned to the intervention and control group by an investigator blinded to the server identification. The allocation sequence was computer generated resulting in five servers (seven healthcare Centers, 58 family physicians) allocated to the intervention group, and four servers (seven healthcare centers, 59 family physicians) allocated to the control group.

To guarantee allocation anonymity, family physicians at each healthcare center only received information about this trial implementation after randomization has been performed. Given the nature of this trial, no consent at physician level was obtained. Consent was obtained from the Northern Regional Health Administration and the Executive Council of the Western Oporto Group of Health Centers.

### Statistical methods

To examine whether the software modification changed test prescription trends we performed an interrupted time series analysis with an autoregressive integrated moving average (ARIMA) model, using the monthly number of tests prescribed per 100 consultations and the intervention (the software modification) as a dichotomous variable (before and after intervention). In the control group, we also performed an ARIMA model analysis using the monthly number of tests prescribed per 100 consultations and the same dichotomous variable (before and after intervention), although there was no intervention in this group. The before and after intervention comparison of the monthly average number of diagnostic and lab tests prescribed per 100 consultations between control and intervention groups was made using an independent sample t test. The Bonferroni correction was used for adjusting for multiple testing (several diagnostic and laboratory tests) [22]. A significance level of 0.05 was used. As size effect measure, Cohen’s d was calculated and a d of ≥0.8 was considered a large effect.

### Ethical considerations

This study was approved by the Northern Regional Health Administration Medical Ethics Committee with several considerations: “1) Given that the study will collect only anonymous data there is no need for the obtainment of informed consent by physicians; 2) The study is of great relevance and with an expected practical interest of the results; and 3) The methodology used safeguards the rights of participants.”

In accordance with the Medical Ethics Committee, before the implementation of the study (but after the randomized allocation), a letter was sent to all Western Oporto Group of healthcare centers informing them of and explaining the study aims and methodology.

## Results

**Fig. 3:**
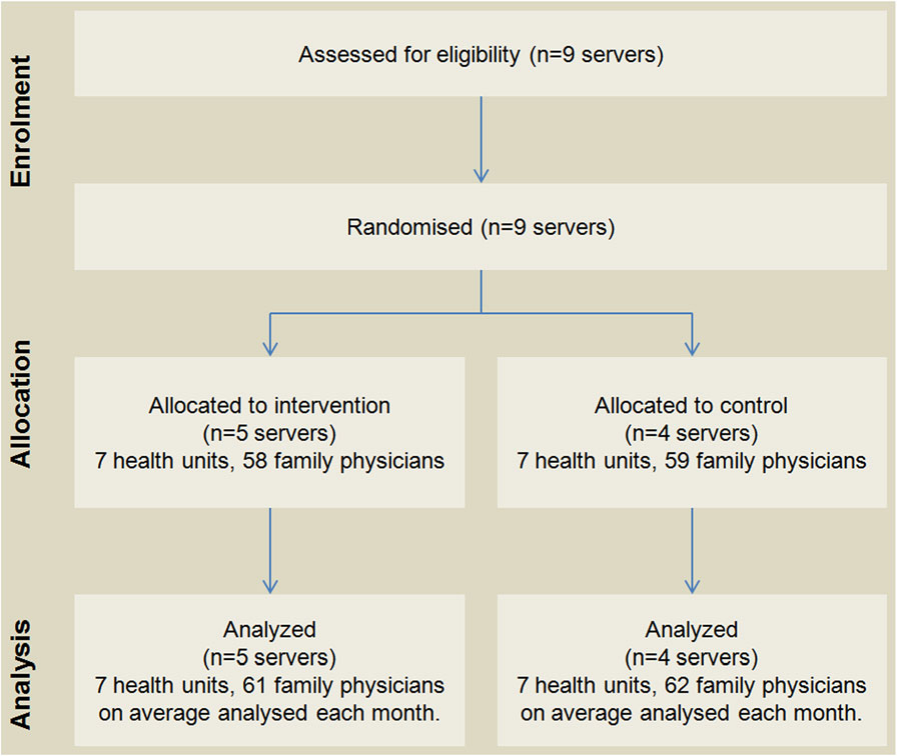
Flowchart summary of the trial

The number of servers enrolled was constant without loss (Fig. 3). The number of family physicians after the intervention ranged from 58 to 64 in the intervention group and from 55 to 70 in the control group. Recruitment was performed in April 2012 and the trial began on 1^st^ June 2012 with a follow-up period until 31^st^ January 2013 (8 month follow-up). Baseline data are presented in Table 2.

**Table 2:** Baseline data ranges for each group based on the last 6 months before the electronic health record (EHR) software modification

	Usual software (control group) min - max	Modified software (intervention group) min - max
Family physicians	57–61	55–63
Total of patients covered by Health Centers	110,781–110,783	79,847–79,850
Face to face consultations	10,725–13,493	10,047–12,598

For both groups, Table 3 compares the monthly average number of diagnostic and laboratory tests prescribed for each 100 consultations before and after EHR software modification, under a time series analysis perspective (ARIMA model). Within the five tests that were withdrawn from the basic menu, we observe a statistically significant reduction in the prescription of four tests (uric acid, serum protein electrophoresis, sedimentation rate, and lung X-ray tests) in the intervention group after software modification. Within the five tests that were added to the basic menu, we observed a significant increase in prescription of the faecal occult blood test in the intervention group after software modification. Within the tests that were marked with green dots (USPSTF recommendation grades A and B) there were no significant increases in either the control or intervention groups. Within the tests that were marked with red dots (USPSTF recommendation grade D), there was a significant reduction only in the prescription of cancer antigen 19-9, but the amount of this test that was prescribed was so low that this variation may be justified by other factors and not a result of software modification.

**Table 3:** Pre-post comparison of the monthly number of diagnostic and lab tests prescribed per 100 consultations (time series analysis, ARIMA model)

	Control group			Intervention group		
	(always exposed to usual EHR software)					
	Before	After	*p*	Before software modification	After software modification	*p*
Diagnostic and lab tests that were withdrawn from the basic menu						
Uric acid	12	10.9	0.094	11.2	4.5	<0.001
Serum protein electrophoresis	4.1	3.2	0.001	4.7	0.6	<0.001
Sedimentation rate	8.3	7.7	0.259	7.1	2	<0.001
Rest electrocardiography^a^	7.7	6.8	0.189	6.4	5.4	0.072
Lung X-ray	1.7	1.6	0.351	1.9	1.4	0.002
All tests withdrawn	33.8	30.3	0.075	31.3	13.9	<0.001
Diagnostic and laboratory tests that were added to the basic menu						
HDL cholesterol^b^	19	17.8	0.357	16.7	16	0.566
Triglycerides	19.6	18.3	0.349	17.4	16.2	0.372
Faecal occult blood test^b^	8.5	7.3	0.236	4.9	6.3	0.017
Cervicovaginal cytology^b^	4.8	4.7	0.779	4.5	4.4	0.905
Mammography^b^	1.9	1.8	0.332	1.6	1.7	0.631
All tests added	53.7	49.9	0.334	45	44.7	0.909
Diagnostic and laboratory tests that were marked with green dots (USPSTF grade A and B)						
Glucose	22.7	21.5	0.402	20.4	18.8	0.199
Total cholesterol	20.6	19.3	0.345	18.3	16.9	0.264
Colonoscopy	1.4	1.5	0.325	1.2	1.2	0.940
Flexible sigmoidoscopy	0.1	0.1	0.031	0.1	0.1	0.115
DXA^α^	1	0.7	0.035	0.6	0.6	0.562
VDRL^β^	2.1	2	0.234	1.9	1.6	0.003
All green marked	82.1	76.7	0.331	70.2	67.5	0.560
Diagnostic and laboratory tests that were marked with red dots (USPSTF grade D)						
Pelvic ultrasound	1.2	1.1	0.033	1	1.1	0.815
Cancer antigen 19-9	0.2	0.2	0.911	0.1	0.1	0.013
Exercise electrocardiography	0.5	0.6	0.308	0.5	0.4	0.113
Carotid artery ultrasound	0.3	0.2	0.484	0.2	0.2	0.072
Spirometry	0.5	0.5	0.959	0.3	0.5	0.010
Hepatitis B surface antigen	1.6	1.7	0.627	1.3	1.2	0.147
Hepatitis C antibodies	1.2	1.2	0.379	0.9	0.9	0.898
All red marked	13.1	12.3	0.341	10.9	9.7	0.102

^a^Tests that were also marked with red dots. ^b^Tests that were also marked with green dots

α- DXA: Dual-energy X-ray absorptiometry β-VDRL: Venereal Disease Research Laboratory

Table 4 presents a direct comparison between the control and intervention groups before and after software modifications (excluding the first month following modification that was considered as a washout period). Regarding the set of tests that were withdrawn from the basic menu, we observe that there were no significant differences between either group before software modification, but that there was a significantly lower prescription rate in the intervention group for the following tests after software modification: 1) uric acid; 2) serum protein electrophoresis; and 3) sedimentation rate. When we consider all tests belonging to the set withdrawn from the basic menu, we have verified that after software modification the intervention group prescribed less than half of those tests when compared with the control group (14.01 tests per 100 consultations versus 29.29 tests per 100 consultations, *p* < 0.001). Regarding the five tests that were added to the basic menu, we observed no significant differences after software modification between the two groups. Considering the tests that were marked with green dots (USPSTF recommendation grades A and B) we observed no significantly higher prescription rates in the intervention group after the software modification compared to the control group. For the tests that were marked with red dots (USPSTF recommendation grade D), we observed significant lower prescription rates in five tests (cancer antigen 19-9, exercise electrocardiography, carotid artery ultrasound, hepatitis B surface antigen, and hepatitis C antibodies) in the intervention group after software modification.

**Table 4:** Comparison of the monthly number of diagnostic and lab tests prescribed per 100 consultations between control and intervention groups, before and after EHR software modification (independent sample t test with Bonferroni correction)

	Before (always exposed to usual EHR software)				After software modification (first month, washout period, excluded)			
	Control	Intervention	*p*	Cohen’s d	Control	Intervention	*p*	Cohen’s d
Diagnostic and lab tests that were withdrawn from the basic menu								
Uric acid	12.0	11.2	1.000	0.632	10.5	4.6	<0.001	6.138
Serum protein electrophoresis	4.1	4.7	0.605	−1.039	3.2	0.6	<0.001	7.384
Sedimentation rate	8.3	7.1	0.315	1.207	7.5	2.0	<0.001	10.818
Rest electrocardiography^a^	7.7	6.4	0.465	1.073	6.6	5.4	0.070	1.544
Lung X-ray	1.7	1.9	0.985	−0.802	1.5	1.4	1.000	0.441
All tests withdrawn	33.8	31.3	0.292	0.641	29.29	14.01	<0.001	7.174
Diagnostic and laboratory tests that were added to the basic menu								
HDL cholesterol^b^	19	16.7	0.640	0.958	17.2	15.9	1.000	0.702
Triglycerides	19.6	17.4	0.830	0.862	17.7	16.1	0.640	0.874
Faecal occult blood test^b^	8.5	4.9	0.015	2.207	6.9	6.2	0.425	1.003
Cervicovaginal cytology^b^	4.8	4.5	1.000	0.468	4.5	4.4	1.000	0.361
Mammography^b^	1.9	1.6	0.855	0.851	1.7	1.7	1.000	0.157
All tests added	53.7	45.0	0.070	1.172	48.0	44.3	0.158	0.805
Diagnostic and laboratory tests that were marked with green dots (USPSTF grade A and B)								
Glucose	22.7	20.4	0.864	0.914	20.9	18.6	0.228	1.246
Total cholesterol	20.6	18.3	0.930	0.885	18.7	16.8	0.450	1.040
Colonoscopy	1.4	1.2	0.042	1.943	1.5	1.1	0.006	2.345
Flexible sigmoidoscopy	0.12	0.07	<0.001	3.002	0.05	0.08	0.186	−1.326
DEXA	1.0	0.6	0.036	2.010	0.7	0.5	0.414	1.063
VDRL	2.1	1.9	0.552	1.077	1.9	1.6	0.102	1.483
All green marked	82.1	70.2	0.070	1.170	74.1	66.8	0.070	1.064
Diagnostic and laboratory tests that were marked with red dots (USPSTF grade D)								
Pelvic ultrasound	1.20	1.04	0.084	1.774	1.02	1.08	1.000	−0.305
Cancer antigen 19-9	0.18	0.13	0.616	1.093	0.17	0.08	<0.001	2.513
Exercise electrocardiography	0.52	0.48	1.000	0.416	0.56	0.38	0.028	1.900
Carotid artery ultrasound	0.27	0.23	1.000	0.750	0.25	0.15	0.021	1.978
Spirometry	0.47	0.34	0.021	2.307	0.45	0.52	0.812	−0.902
Hepatitis B surface antigen	1.60	1.34	0.084	1.756	1.59	1.24	0.028	1.925
Hepatitis C antibodies	1.15	0.91	0.021	2.207	1.18	0.91	0.014	2.045
All red marked	13.1	10.9	0.021	1.583	11.8	9.8	0.003	1.941

^a^Tests that were also marked with red dots. ^b^Tests that were also marked with green dots

*α- DXA* Dual-energy X-ray absorptiometry, *β-VDRL* Venereal Disease Research Laboratory

## Discussion

Our results show that removing unnecessary tests from a quick shortcut menu of diagnostic and laboratory tests available on the EHR test ordering system might have significantly impacted ordering habits and reduced unnecessary test prescriptions. This result emphasized the importance of careful attention and scientific rigor when building shortcut menus in ordering systems. This impact was significant, and it could be observed from two perspectives of analysis: 1) when we compared the results for the intervention group before and after the modification of the software and 2) when we compared the intervention group with the control group.

Furthermore, our results demonstrated that the introduction of an evidence-based decision support tool had no significant impact on the test prescription profiles. However, it should be noted that as a passive instrument, the decision for support addition did not interfere with the normal flow of the ordering system. The doctors had only visual contact with the colored recommendations and optional access to the additional information from the USPSTF recommendations. The absence of impact of this support decision tool may also be related to high level of agreement between the way Portuguese family doctors perform preventive health services and the USPSTF recommendations as shown in a previous national cross-sectional study [22].

Limitations of our study include the sampling method and sample size. The moderate size of the sample might have limited our conclusions regarding the less frequently prescribed tests, but this is unlikely to have affected the main conclusion of our study.

The fact that it was not possible to perform randomization at the family physician level, but only at that of the computer servers, also constitutes a limitation and a possible biasing factor, since the number of servers in the Western Oporto Grouping of healthcare centers was small. In the Western Oporto Grouping of healthcare centers we had nine servers. Since our intervention was a software modification, we had to randomize at the level of the electronic server. One hypothesis would be used to consider each server as a cluster, but the problem is that some servers serve only one health unit, not being a real cluster, while others serve two or three healthcare units. The ideal would be to consider each healthcare unit as a cluster of family physicians. For that, however, we had to have access to the number of tests prescribed by each family physician, which we did not. The Ministry of Health only allowed us access to the tests prescribed by each healthcare unit. This is the reason why it is not possible to consider this study as a cluster randomized controlled trial. Without the number of tests prescribed by each family physician, we were not able to calculate the intracluster correlation coefficient.

Another limitation of our study is that the time available for its completion was relatively short. An extended study period could have allowed us to draw more extensive conclusions (for example, regarding the tests that are prescribed less frequently). A prolonged study period would also have diluted possible seasonal effects that might exist in seeking medical consultations and also minimized the impact of factors inherent in the economic situation of Portugal during the study period. In the context of the economic crisis, there was an increase in user fees paid by patients for medical consultations and laboratory tests in addition to Ministry of Health publications of clinical guidelines for prescriptions of some diagnostic and laboratory tests. These facts may have contributed to the gradual decrease in test prescriptions that we have observed during the study period both in the control and intervention groups. The extension of the study might also have diluted this effect and could have allowed a clearer reading of the impact of software modifications. However, the socio-economic status of intervention and control medical centers was identical. All healthcare centers involved in the study are located in the urban area of Oporto in a geographic area of approximately 22Km^2^, [23]. All study physicians were exposed to the same Ministry of Health guidelines and general education materials.

These limitations were mainly due to technical issues related to the fact that our research could not disrupt the normal functioning of resources and services of the Ministry of Health. However, we consider it unlikely that any of these limitations limited the main conclusion of this study: the considerable impact of unnecessary test removal in the shortcut menu of the ordering system.

Considering the potential generalizability of our results to other groups of healthcare centers of the Portuguese National Health Service, we believe that there is a high probability that the same effect will be found with similar software intervention. We believe that this is the case for two reasons: 1) the size of the obtained effect was evident and 2) the main features of the ordering system of other groups of healthcare centers are similar.

For this study, we used the recommendations of the USPSTF as our standard guidelines because it bases its recommendations on freely accessible, evidence-based systematic reviews with recognized methodological quality that covers a considerable range of topics. Portugal is in a phase of transition regarding clinical guidelines and recommendations. The Ministry of Health has recently published guidelines covering some topics related with test prescriptions. However, many of these guidelines are still in the phase of public discussion and are not based on systematic reviews but rather on experts’ opinion. For these reasons we chose the USPSTF recommendations. Exceptions to this are the recommendations of the Portuguese Ministry of Health for three tests: 1) breast cancer screening by mammography every two years for women 50**–**69 years old; 2) colorectal cancer screening by a faecal occult blood test every 1–2 years for adults 50–74 years old; and 3) cervical cancer screening with a cervicovaginal cytology for women between 25 and 60 years every three years after two annual normal tests [24]. These recommendations are in line with those from USPSTF but could have influenced our results.

When designing this trial the researchers also considered the possibility of causing some negative effects as a result of the intervention. There could be two main type of negative effects: 1) on physicians because they could not find the tests in the basic shortcut menu as they previously had and it would consume more consultation time to type the name in the search box to order one of the removed tests and 2) on patients because they could be deprived of some necessary tests. After debating this issue with some family physicians, these potential negative effects were considered negligible, mainly because there was no interference with physicians’ freedom to prescribe any medical test and the use of the search box as an alternative to the shortcut menu was not considered a major obstacle.

The reduction of unnecessary test prescriptions that we verified was in line with other studies in which changes in laboratory request forms also resulted in the reduction of unnecessary tests requests [25–27]. However, there are some practical implications that may result from this study. For example, the Portuguese Health Authorities could use these results to improve test ordering software and to reduce the prescription of unnecessary tests at a national level. This study also reinforces the importance of adequately testing medical related software tools, and we hope that in the future, this will be the rule for other medical software tools.

## Conclusions

Our results might have a significant impact on improving the design of shortcut menus of diagnostic and laboratory test ordering systems either in the Portuguese National Health Service or in other countries’ health systems. These improvements can help reduce the prescription of unnecessary tests, leading to the reduction of negative patient effects and to the reduction of unnecessary costs. These study results demonstrate the importance of testing and evaluating various aspects of medical informatics programs to improve efficiency and contribute to improved clinical practice and clinical outcomes.

## Abbreviations

ARIMA: Autoregressive integrated moving average
HER: Electronic health records
SAM: *Sistema de Apoio ao Médico* (Physician’s Support System)
USPSTF: United States Preventive Services Task Force
